# Genotype and Haplotype Analyses of *TP53* Gene in Breast Cancer Patients: Association with Risk and Clinical Outcomes

**DOI:** 10.1371/journal.pone.0134463

**Published:** 2015-07-30

**Authors:** Veronika Vymetalkova, Pavel Soucek, Tereza Kunicka, Katerina Jiraskova, Veronika Brynychova, Barbara Pardini, Vendula Novosadova, Zdena Polivkova, Katerina Kubackova, Renata Kozevnikovova, Miloslav Ambrus, Ludmila Vodickova, Alessio Naccarati, Pavel Vodicka

**Affiliations:** 1 Institute of Experimental Medicine, Academy of Sciences of the Czech Republic, Prague, Czech Republic; 2 Institute of Biology and Medical Genetics, First Faculty of Medicine Charles University, Prague, Czech Republic; 3 Toxicogenomics Unit, National Institute of Public Health, Prague, Czech Republic; 4 Human Genetics Foundation, Turin, Italy; 5 Institute of Biotechnology, Academy of Sciences of the Czech Republic, Prague, Czech Republic; 6 Department of Oncology, University Hospital Motol, Prague, Czech Republic; 7 Department of Oncosurgery, MEDICON, Prague, Czech Republic; 8 Department of Radiotherapy and Oncology, Third Faculty of Medicine, Charles University, Prague, Czech Republic; 9 Department of General Biology and Genetics, Third Faculty of Medicine, Charles University, Prague, Czech Republic; 10 Third Faculty of Medicine, Charles University, Prague, Czech Republic; University of Texas MD Anderson Cancer Center, UNITED STATES

## Abstract

Variations in the *TP53* gene have been suggested to play a role in many cancers, including breast. We previously observed an association between *TP53* haplotypes based on four polymorphisms (rs17878362, rs1042522, rs12947788, and rs17884306) and the risk of colorectal and pancreatic cancer. Based on these results, in the present study, we have investigated the same polymorphisms and their haplotypes in 705 breast cancer cases and 611 healthy controls in relation to the disease risk, histopathological features of the tumor and clinical outcomes. In comparison to the most common haplotype A_1_-G-C-G, all the other identified haplotypes were globally associated with a significantly decreased breast cancer risk (P = 0.006). In particular, the A_2_-G-C-G haplotype was associated with a marked decreased risk of breast cancer when compared with the common haplotype (P = 0.0001). Moreover, rs1042522 in patients carrying the GC genotype and receiving only the anthracycline-based chemotherapy was associated with both overall and disease-free survival (recessive model for overall survival HR = 0.30 95% CI 0.11–0.80, P = 0.02 and for disease-free survival HR = 0.42 95% CI 0.21–0.84, P = 0.01). Present results suggest common genetic features in the susceptibility to breast and gastrointestinal cancers in respect to *TP53* variations. In fact, similar haplotype distributions were observed for breast, colorectal, and pancreatic patients in associations with cancer risk. Rs1042522 polymorphism (even after applying the Dunn-Bonferroni correction for multiple testing) appears to be an independent prognostic marker in breast cancer patients.

## Introduction

Breast cancer (BC) is the third most common tumor worldwide (and the most frequent in women), representing 9% of the global cancer burden. Epidemiologic studies have suggested a number of risk factors including personal, family, and gynecological history, as well as an interplay between genetic and environmental factors [1]. Mutations in the rare high penetrance BC predisposing genes *BRCA1* and *BRCA2* account for 16–25% of the inherited component of this cancer [2]. To date, GWAS conducted for BC have identified more than 80 breast cancer susceptibility loci (all summarized in [3]). Although, several candidate gene association studies for BC have been conducted during the last decade [4–10], many of them were underpowered due to small sample size, resulting in inconsistent and not reproducible findings [11,12]. To date, only one polymorphism located in the coding region of *CASP8* (rs1045485) has shown promise as a breast cancer predisposition risk factor [3,13].

The prognosis of breast carcinoma is mostly influenced by tumor stage, grade, overexpression of v-erb-b2 avian erythroblastic leukemia viral oncogene homolog 2 (*ERBB2*), and hormonal receptors. These factors are routinely considered in selecting the treatment regimen for each individual [14]. Anthracycline combination chemotherapies are the most effective and widely used regimens for the treatment of BC [15]. Radiotherapy, on the other hand, improves BC survival by reducing the risk of local recurrence, whereas it has no direct impact on metastasis [16]. Besides, still novel biomarkers are needed to identify individuals who would benefit most from a given treatment regimen. Such markers might arise from inherited genetic variation, which—apart from BC susceptibility—may affect disease progression, response to treatment and outcome [17].

p53 protein (encoded by *TP53* gene) is a key player in stress responses that preserve genomic stability responding to a variety of insults including DNA damage, hypoxia, metabolite stress and oncogene activation [18]. In addition, p53 interacts with numerous cellular proteins including those controlling the programmed cell death. This tumor suppressor gene is frequently mutated in various solid tumors, including colorectum, pancreas, and breast. These mutations result in the absence or dysfunction of the corresponding protein [19]. *TP53* is also polymorphic: so far, 547 single nucleotide polymorphisms (SNPs) have been identified (http://genecards.org). Owing to the importance of p53 in tumor suppression, SNPs that may alter its function might affect not only cancer risk but also its progression and/or response to the treatment [18]. *TP53* rs1042522 is one of the most commonly investigated variants in cancer genetic epidemiology. However, two recent reviews [20,21] suggested that this polymorphism is unlikely to be a risk factor for BC. Instead, a recent meta-analysis on rs17878362 (PIN3, Ins11951_11966, allele A2 carries the 16-bp insertion within intron 3) has found an increased risk of BC in A_2_A_2_ carriers [22]. Previous studies have suggested rs1042522 as a prognostic marker for BC [23–26]. On the contrary, the role of *TP53* haplotypes has not yet been fully investigated for BC susceptibility and its clinical outcome.

Previously, we have studied the role of four *TP53* polymorphisms on colorectal cancer (CRC) and pancreatic cancer risk. In both studies, we have observed a consistent differential distribution of the major haplotypes built on the four variants between cases and controls, suggesting that prevalent haplotypes within the *TP53* gene may modulate both CRC and pancreatic cancer risk [27,28]. In the present investigation, we evaluated the association of the same variations within *TP53* gene and resulting haplotypes (loci in the order: rs17878362, rs1042522, rs12947788, and rs17884306) with the risk of BC in a hospital-based case-control study from the Czech Republic. In this country, around 6,000 women every year are diagnosed with BC, and about 2,000 women die because of this cancer (incidence rank: 30th worldwide and 18th in Europe; mortality rates rank: 117^th^ worldwide and 36th in Europe; www.mamo.cz). In a set of the BC patients with complete follow-up, we also investigated the prognostic relevance of *TP53* gene variability.

## Material and Methods

### Study population

The study population comprised 705 BC patients and 611 healthy controls. Blood samples were obtained from women with incident BC consecutively diagnosed in three hospitals in Prague between February 2002 and December 2010. Sample collection from patients was described elsewhere [29–31]. All subjects were informed and provided written consent to participate in the study and to use their biological samples for genetic analyses, according to the Helsinki declaration. The design of the study was approved by the Ethical Committee of the Institute of Experimental Medicine, Prague, Czech Republic.

The control group consisted of healthy women whose information were previously described in other association studies [27,32–34]. Two control groups were included in the study. The first group consisted of 268 hospital-based individuals admitted to gastroenterological departments that had negative colonoscopy results for malignancy or idiopathic bowel diseases. The reasons for undergoing the colonoscopy were: (i) positive fecal occult blood test, (ii) hemorrhoids, (iii) abdominal pain of unknown origin, and (iv) macroscopic bleeding. They did not have any malignancy at the time of the sampling. The second group of controls consisted of 343 healthy blood donor volunteers collected from a blood donor center in Prague. All individuals were subjected to standard examinations to verify the health status for blood donation and were cancer-free at the time of the sampling.

The following data on patients were retrieved from medical records: date of cancer diagnosis, age, menopausal status, family history of cancer (number of relatives affected by BC, ovarian cancer or other malignant diseases), tumor size, UICC (International Union Against Cancer) tumor-node-metastasis (TNM) system classification, histological type and grade of tumor, expression of estrogen receptor (ER), progesterone receptor (PR) and ERBB2, expression of the Ki-67 protein, chemotherapy and hormonal regimen. ERBB2 status was defined as positive in samples with an immunohistochemical score of 2+ or 3+ [35]. Due to different treatment of patients with metastasis, those women with metastasis were excluded from the analysis.

For all cases, detailed information on radiotherapy, first line chemotherapy, and hormonal therapy was available. Patients were treated with chemotherapy regimens containing 5-fluorouracil, anthracycline, cyclophosphamide, and/or taxanes (for all treatments see **S1 File**). Information about distant metastasis, relapse and date of death was also collected, with a follow-up until December 31, 2013. In this study, the outcome variables measured were overall survival (OS) and disease-free survival (DFS). The same variables were also investigated after stratification according to anthracycline-, taxane-based chemotherapy, or hormonal-based therapy,

### Selection of polymorphisms

The *TP53* SNPs analysed were the same as those previously investigated for CRC [27] and pancreatic cancer [28] studies and represent part of the genetic variation in *TP53* gene with MAF≥5%. In particular, three tag SNPs were selected (rs1042522:G>C (Ex4+119 G>C, Arg72Pro), rs12947788:C>T (IVS7+72 C>T) and rs17884306:G>A (Ex11-363 G>A)). In the study, we also included the 16 bp insertion/deletion polymorphism within the intron 3 (rs17878362:A1>.A2, PIN3, Ins11951_11966, in which the A2 allele carries the 16 bp insertion within the intron 3).

### SNP Genotyping

Genomic DNA was isolated from peripheral blood lymphocytes using standard procedures as described in [36]. DNA samples from cases and controls were randomly placed on plates where an equal number of cases and controls were run simultaneously. Genotyping of the selected SNPs was carried out by using the KASPar chemistry of LGC Genomics (http://www.lgcgenomics.com/genotyping/kasp-genotyping-reagents/kasp-technical-resources/), as described in [37]. Duplicate samples (5%) and non-template controls in each plate were used as quality control tests.

Rs17878362 in *TP53* gene was genotyped by PCR-RFLP as previously described in [27] and [28].

### Statistical analyses

Chi-square test (1 degree of freedom), with a type-I error threshold set at α = 0.05, was used to verify whether the genotypes were in Hardy-Weinberg equilibrium in controls. The haplotype frequencies in cases and controls were estimated with the SAS/Genetics software module (SAS Institute, Cary, NC, USA). The analysis was carried out to examine the phase of *TP53* SNPs using the expectation–maximization algorithm to generate maximum likelihood estimates of haplotype frequencies.

The association between SNPs and BC risk was calculated by estimating the odds ratios (ORs) and their 95% confidence intervals (CI) adjusted for age. For all SNPs, the co-dominant, the dominant or the recessive models were calculated.

OS was defined as the time from the surgery to the date of death or the date of the end of the study (December 31, 2013). DFS was defined as the time from surgery/end of therapy to the occurrence of distant metastasis, local recurrence or death, whichever came first. The relative risk of death and recurrence was estimated as hazard ratio (HR) using Univariate Cox regression, Multivariate Cox regression for significant results and Long-rank test. The survival curves for overall and disease-free survival were derived by the Kaplan–Meier method. Multivariate survival analyses were adjusted for age and therapy. Statistical analyses were performed using SAS software (SAS Institute, Cary, NC, USA).

## Results

### Case-control study

The study included 705 cases and 611 controls (detailed information in **S1 File**). The median age (range) of patients at diagnosis was 59 (65–92) years while the median age of controls at recruitment was 49 (57–85) years. There was a statistically significant difference in the age distribution between BC patients and controls (P<0.0001). Due to differences in age among cases and controls, we have considered this factor as a confounding variable and used in our analysis adjusting OR for it.

The distribution of genotypes within the four selected *TP53* SNPs in controls was in agreement with the Hardy-Weinberg equilibrium (**Table 1**). No significant differences were found among cases and controls in the genotype frequencies for any of the polymorphisms when analyzed individually (**Table 1**). Rs17884306 resulted monomorphic (only GG genotype in all BC subjects). Additionally, no clinicopathological parameters were associated with the risk of BC after both univariate and multivariate analysis.

**Table 1:** Genotype distribution of investigated *TP53* polymorphisms in BC patients and controls.

Genotypes	Controls^a^	Cases^a^	OR^b^	95% CI	P	HWE^c^
	(n = 611)	(n = 705)				Χ^2^, P
**rs17878362** ^**d**^						0.01, 0.99
*A*_*1*_ *A*_*1*_	421	474	REF			
*A*_*1*_ *A*_*2*_	172	164	0.88	0.67–1.16	0.36	
*A*_*2*_ *A*_*2*_	18	24	1.13	0.57–2.24	0.71	
*A*_*1*_ *A*_*2*_ *+ A*_*2*_ *A*_*2*_	190	188	0.91	0.70–1.18	0.46	
**rs1042522**						0.35, 0.84
*GG*	301	370	REF			
*GC*	260	275	0.89	0.70–1.14	0.35	
*CC*	50	55	0.93	0.61–1.43	0.74	
*GC+CC*	310	330	0.90	0.71–1.14	0.37	
**rs12947788**						0.24, 0.89
*CC*	530	594	REF			
*CT*	78	102	1.26	0.90–1.77	0.19	
*TT*	2	5	1.60	0.30–8.53	0.58	
*CT+ TT*	80	107	1.27	0.91–1.77	0.16	
**rs17884306**						0.48, 0.79
*GG*	540	702				
*GA*	66	0	-	-	-	
*AA*	1	0	-	-	-	
*GA+AA*	67	0	-	-	-	

^a^Numbers may not add up to 100% of subjects due to genotyping failure. All samples that did not give a reliable result in the first round of genotyping were resubmitted to up to two additional rounds. Data points that were still not filled after this procedure had been left blank.

^b^Logistic regression analysis values are adjusted for age.

^c^X^2^ and P-values for the deviation of observed and the numbers expected from the Hardy-Weinberg equilibrium (HWE) in the controls.

^d^Allele A_2_ carries the 16-bp insertion within intron 3.

OR, odds ratio; CI, confidence interval.

In the present study, the two most frequent haplotypes A_1_-G-C-G and A_2_-C-C-G comprised the 83% of cases and only the 77% of controls. The A_2_-G-C-G haplotype was associated with a significantly decreased risk of BC when compared with the reference haplotype A_1_-G-C-G, comprising only the common alleles (OR = 0.36, 95%CI 0.21–0.61, P = 0.0001; **Table 2**). When the reference haplotype A_1_-G-C-G was compared to all other haplotypes, it resulted in association with an increased BC risk (OR = 1.29, 95%CI 1.08–1.54, P = 0.006).

**Table 2:** *TP53* haplotype distribution between BC patients and controls.

Haplotypes^a^	Controls^b^	Cases^b^	OR^c^	95% CI	P
	n = 1222	n = 1410			
^**d**^ **A**_**1**_ **-G-C-G**	775	898	REF		
**All others**	437	394	0.78	0.65–0.93	0.006*
**A**_**2**_ **-C-C-G**	153	179	1.06	0.82–1.37	0.67
**A**_**1**_ **-C-C-G**	84	85	0.81	0.58–1.13	0.21
**A**_**1**_ **-C-T-G**	65	95	1.36	0.96–1.93	0.09
**A**_**1**_ **-C-C-A**	55	0	-	-	-
**A**_**2**_ **-G-C-G**	50	24	0.36	0.21–0.61	0.0001*
**A**_**1**_ **-G-T-G**	17	8	-	-	-
**A**_**1**_ **-G-C-A**	9	0	-	-	-
**A**_**2**_ **-G-C-A**	3	0	-	-	-
**A**_**2**_ **-C-C-A**	1	0	-	-	-
**A**_**2**_ **-C-T-G**	0	2	-	-	-
**A**_**2**_ **-G-T-G**	0	1	-	-	-

^a^Loci rs17878362, rs1042522, rs12947788, rs17884306.

^b^Number of alleles are reported. Because each individual has two alleles, the total number of alleles will be twice the total number of individuals. Individuals with missing haplotype data were not included in the analyses.

^c^Adjusted for age.

^d^Allele A_2_ carries the 16-bp insertion within intron 3

OR, odds ratio; CI, confidence interval. Significant P-values are in bold.

During the follow-up of BC cases, overall 77 patients died, with 39 of them due to cancer progression. In addition, 78 subjects had recurrence (**S1 File**). Forty four patients were not retrievable during follow-up, therefore, they were excluded from the analysis. The median OS and DFS for the studied population were 54.3 and 52.2 months, respectively.

One hundred forty-four (20.4%) patients received neoadjuvant chemotherapy before surgery. Concerning the adjuvant chemotherapy, 192 (51.9%) patients were administered with antracyclines, 93 (25.1%) with taxanes, 34 (9.2%) with both agents in combination and 44 (11.9%) with others agents as first-line postoperative therapy (for more detail see **S1 File**). Two hundred ninety-one (41.3%) subjects did not receive any adjuvant chemotherapy after surgery, while 218 (30.9%) women received the hormonal therapy as a first line postoperative therapy instead of adjuvant therapy. Finally, 318 (45.1%) patients were administrated with both adjuvant and hormonal therapy, comprising mainly tamoxifen or inhibitors of aromatases. Patients not receiving any hormonal therapy or radiotherapy or with advanced age (>60 years) resulted to have a shorter OS (log-rank P = 0.04; P = 0.006 and P = 0.01, respectively) and DFS (log-rank P = 0.04; P = 0.002 and P = 0.02, respectively) when compared with those receiving hormonal or radiotherapy or with age lower than 60, respectively. No other clinicopathological parameters were associated with survival (after both univariate and multivariate analysis).

Patients with homozygous variant genotype A_2_A_2_ of rs17878362 polymorphism were at higher risk of relapse or metastasis (HR = 2.15, 95%CI 1.04–4.44, P = 0.04; **Table 3**). On the other hand, patients carrying GC genotype of rs1042522 were at lower risk of relapse or metastasis (HR = 0.66, 95%CI 0.44–1.00, P = 0.05). Concerning haplotype analyses, the haplotype A_1_-C-C-G was associated with lower risk of relapse or metastasis (HR = 0.34, 95%CI 0.14–0.84, P = 0.02; **Table 4**).

**Table 3:** Overall (OS) and disease-free (DFS) survival in relation to SNP distributions (Cox regression).

Genotype	OS					DFS			
	Cases	Events	HR	95% CI	P	Events	HR	95%CI	P
**rs17878362**									
*A*_*1*_ *A*_*1*_	470	52	REF	-	-	78	REF	-	-
*A*_*1*_ *A*_*2*_	163	16	0.87	0.50–1.52	0.63	24	0.88	0.56–1.39	0.58
*A*_*2*_ *A*_*2*_	24	3	1.07	0.33–3.43	0.91	8	2.15	1.04–4.44	**0.04**
*A*_*1*_ *A*_*2*_ ***+*** *A*_*2*_ *A*_*2*_	187	19	0.90	0.53–1.52	0.69	32	1.03	0.68–1.56	0.88
**rs1042522**									
*GG*	369	44	REF	-	-	71	REF	-	-
*GC*	271	25	0.82	0.50–1.34	0.42	33	0.66	0.44–1.00	**0.05**
*CC*	55	4	0.62	0.22–1.71	0.35	9	0.88	0.44–1.75	0.71
*GC+CC*	326	29	0.78	0.49–1.25	0.30	42	0.70	0.48–1.02	0.06
**rs12947788**									
*CC*	592	63	REF	-	-	97	REF	-	-
*CT*	99	11	1.06	0.56–2.00	0.87	16	1.00	0.59–1.70	0.99
*TT*	5	0	-	-	-	0	-	-	-
*CT+TT*	104	11	1.00	0.53–1.90	0.99	16	0.95	0.56–1.61	0.85

The SNP rs17884306 was monomorphic in cases, thus not presented.

HR, hazard ratio; 95% CI, confidence interval. Significant results in bold.

**Table 4:** OS and DFS in relation to haplotype distributions (Cox regression).

Haplotype^a^	OS					DFS			
	Cases^b^	Events^b^	HR	95%CI	P	Events	HR	95%CI	P
^**c**^ **A**_**1**_ **-G-C-G**	896	104	REF			157	REF		
**All others**	390	36	0.81	0.55–1.18	0.26	59	0.88	0.65–1.19	0.40
**A**_**2**_ **-C-C-G**	178	16	0.78	0.46–1.32	0.35	29	0.95	0.64–1.42	0.81
**A**_**1**_ **-C-T-G**	92	10	0.97	0.50–1.85	0.91	15	0.96	0.57–1.64	0.89
**A**_**1**_ **-C-C-G**	85	5	0.54	0.22–1.31	0.17	5	0.34	0.14–0.84	0.02*

^a^Loci rs17878362, rs1042522, rs12947788, rs17884306.

^b^ Number of alleles are reported. Because each individual has two alleles, the total number of alleles is twice the total number of individuals. Individuals with missing haplotype data were not included in the analyses.

^c^Allele A_2_ carries the 16-bp insertion within intron 3

HR, hazard ratio; 95% CI, confidence interval. Significant results in bold.

For significant associations, we have also applied the multivariate Cox regression analysis. Patients with advanced age (>60 years old) and those not receiving any hormonal or radiotherapy resulted in association with a shortened OS and DFS (**S1 File**). In the recessive model, rs1042522 was still associated with lower risk of relapse or metastasis (HR = 0.65, 95%CI 0.44–0.96, P = 0.03).

### Adjuvant chemotherapy

**Fig 1:**
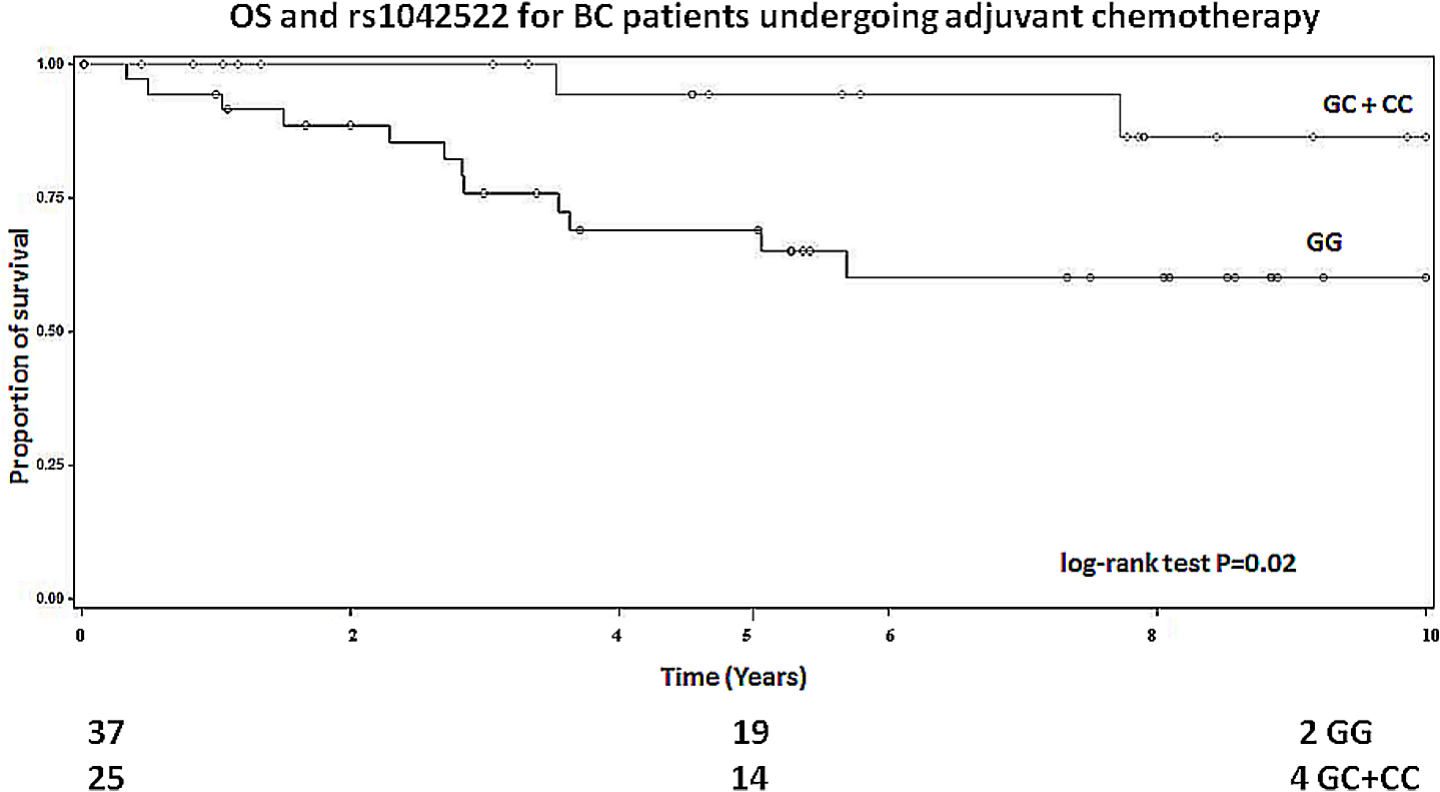
Kaplan-Meier overall survival curves for *TP53* rs1042522 polymorphism in patients receiving only adjuvant chemotherapy (log-rank for recessive model P = 0.02). Numbers of patients at risk are indicated in the lower part of the plot.

In patients receiving only adjuvant chemotherapy, rs1042522 was significantly associated with OS (log-rank for recessive model P = 0.02; **Fig 1**). In particular, when patients with the homozygous GG genotype were compared to C allele carriers, they showed longer survival (HR = 0.21, 95%CI 0.05–0.94, P = 0.04).

After stratification for anthracycline-based chemotherapy, rs17878362 was associated with OS (log-rank P = 0.02). In particular, a better survival was observed for those patients carrying the A_1_A_2_ genotype and undergoing anthracycline-based therapy (HR = 0.14, 95%CI 0.02–1.00, P = 0.05; **Table 5**). In the same group of treated patients, rs1042522 polymorphism was also associated with both OS and DFS (log-rank P = 0.02 and 0.007, respectively; **Table 5**). Namely, we have observed a better survival for those patients carrying the GC genotype and undergoing anthracycline-based chemotherapy (OS: HR = 0.31, 95%CI 0.11–0.90, P = 0.03; DFS: HR = 0.34, 95%CI 0.15–0.77, P = 0.01 respectively). In a recessive model, the associations remained the same (log-rank P = 0.01 and 0.01, respectively; **Table 5).**

**Table 5:** OS and DFS in relation to SNP distributions in patients treated with anthracycline-based chemotherapy (Cox regression).

Genotype		OS				DFS			
	Cases	Events	HR	95% CI	P	Events	HR	95%CI	P
**rs17878362**									
*A*_*1*_ *A*_*1*_	159	22	REF	-	-	35	REF	-	-
*A*_*1*_ *A*_*2*_	53	1	0.14	0.02–1.00	**0.05**	5	0.42	0.16–1.06	0.07
*A*_*2*_ *A*_*2*_	7	2	1.86	0.44–7.93	0.40	3	2.03	0.62–6.61	0.24
*A*_*1*_ *A*_*2*_ ***+*** *A*_*2*_ *A*_*2*_	60	3	0.35	0.11–1.18	0.09	8	0.60	0.28–1.28	0.19
**rs1042522**									
*GG*	120	21	REF	-	-	33	REF	-	-
*GC*	82	4	0.31	0.11–0.90	**0.03**	7	0.34	0.15–0.77	0.01*
*CC*	20	1	0.29	0.04–2.15	0.23	4	0.73	0.26–2.07	0.56
*GC+CC*	102	5	0.30	0.11–0.80	0.02*	11	0.42	0.21–0.84	0.01*
**rs12947788**									
*CC*	189	21	REF	-	-	34	REF	-	-
*CT*	33	5	1.44	0.54–3.82	0.47	10	2.00	0.98–4.00	0.06
*TT*	2	0	-	-	-	0	-	-	-
*CT+TT*	35	5	1.33	0.50–3.53	0.57	10	1.80	0.89–3.65	0.10

The SNP rs17884306 was monomorphic in cases, thus not presented.

HR, hazard ratio; 95% CI, confidence interval. Significant results in bold; significant differences after Dunn–Bonferroni correction (P<0.02) are marked with an asterisk.

The stratification for anthracycline-based chemotherapy or for taxane-based chemotherapy did not show any significant associations of *TP53* haplotype distribution with the OS or DFS (data not shown).

### Hormonal therapy

**Fig 2:**
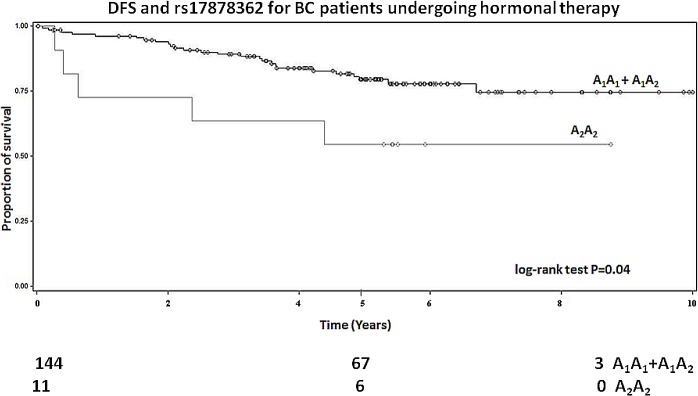
Kaplan-Meier disease-free survival curves for *TP53* rs17878362 polymorphism in patients receiving only the hormonal therapy (log-rank for recessive model). Numbers of patients at risk are indicated in the lower part of the plot.

Among patients receiving only the hormonal therapy, rs17878362 polymorphism was significantly associated with DFS (log-rank for recessive model P = 0.04; **Fig 2**). In particular, we have observed a worse survival for those patients carrying the variant A_2_A_2_ genotype (HR = 2.66, 95%CI 1.02–6.94, P = 0.05).

Further stratification for hormonal-based therapy and the regime containing either tamoxifen or inhibitors of aromatases agents did not show any significant associations with BC prognosis (data not shown).

## Discussion

In the present study, we investigated the role of genetic variations within *TP53* gene on BC risk and its clinical outcomes in a population from the Czech Republic. Recent meta-analyses suggested that polymorphisms in *TP53* taken individually are not presumably risk factors for BC [20,21]. Our data on single *TP53* polymorphisms are in good agreement with the above postulation. In contrast, there are also meta-analyses reporting that rs17878362 may contribute to susceptibility of BC [22,38]. On the other hand, *TP53* haplotypes have not been comprehensively explored regarding the BC risk and prognosis.

Interestingly, in the case-control study we found that the *TP53* A2-G-C-G haplotype was associated with a decreased risk of BC when compared with the most common haplotype A1-G-C-G. Globally, all the detected haplotypes were associated with decreased risk of the disease when compared with A1-G-C-G.

Although some studies found an association between sporadic BC and rs1042522, rs17878362 or their respective haplotypes [39–41], the results are rather inconclusive due to limited sample size (108, 159, 122 patients, respectively), differences in allele frequencies between ethnic groups or staging of the malignancy.

In previous reports, we have observed a differential distribution of specific haplotypes between cancer patients and controls, suggesting that prevalent haplotypes within *TP53* may modulate both CRC and pancreatic cancer risk [27,28]. Interestingly, we have observed a similar pattern also for BC patients. The haplotypes within *TP53* along with SNPs in other genes in its pathway may impact the onset of pancreatic cancer and CRC, as well as BC. Thus, our results seem to point to general mechanisms based on common genetic background involved in the development of solid tumors. As previously observed for colorectal and pancreatic malignancies, the different distribution of *TP53* haplotypes also in BC cases and controls may be due to a linkage of the disease to yet unknown functional polymorphism(s) within *TP53* or in some neighboring genes on 17p that could carry putative functional variant(s) linked to the detected haplotypes. This argument is supported by predictions of such associations and the existence of large haplotype blocks within the human genome [42]. On the other hand, there are several studies in which deletion of 17p have been frequently observed in BC patients [43–45]. The reversal of the modulation effect of *TP53* polymorphisms within haplotypes may also point to additional polymorphism(s) that cause differential cancer risk, either directly or through interaction with environmental factors [27]. p53 interacts with numerous cellular proteins, including those in the control of programmed cell death. Above molecular interactions might contribute to its inhibitory role in the tumorigenesis. *TP53* is frequently mutated in various solid tumors, including colorectal, pancreatic and breast cancers, and these mutations result in the absence or dysfunction of the p53 protein [46].

Another novel finding emerging from this study was that two individual SNPs in *TP53* (rs1042522 and rs17878362) were associated with patient´s survival in a set of 705 BC cases. In particular, patients with variant genotype A_2_A_2_ in rs17878362 were at higher risk of relapse or metastasis. After stratification for anthracycline-based chemotherapy, rs17878362 was also associated with OS. The association of SNPs with longer OS and DFS, particularly in relation to the anthracycline–based chemotherapy, is of interest since it is in contrast to mutations in this gene that are generally associated with a poor prognosis in BC patients [47,48]. Apart from few studies [23,49–51], the effect of *TP53* polymorphisms on patients survival has not been studied in detail. The role of *TP53* as a predictive biomarker for treatment response still remains a matter of debate. Interestingly, [49] observed on a relatively large set of Finnish samples that patients carrying the homozygous variant CC genotype of rs1042522 had significantly poorer survival than BC patients with other genotypes. A similar pattern of association was also observed in the study of Toyama et al. (2007) on Japanese BC women, in the one on a Chinese population [24] and more recently in the work of [26] on a Spanish group of BC. Interestingly, patients with rs1042522 GC genotype were at lower risk of relapse or metastasis, as well as those with the less frequent haplotype A_1_-C-C-G.

The association of rs17878362 to patient’s survival is of particular interest since the functional relevance of rs17878362 is unknown. Intronic sequences in this gene have been implicated in the regulation of gene expression and in DNA–protein interactions [52]. Rs17878362 may also be in linkage disequilibrium with other yet unidentified genes, thus explaining its association with a distinctive phenotype.

The common G allele of rs1042522 is associated with a form of the p53 protein that is a more potent inducer of apoptosis than the one containing the C allele [53]. It has been suggested that patients carrying the GG genotype may respond more favorably to radiation or chemotherapy [54]. For example, the higher efficiency of GG genotype in inducing apoptosis is reflected *in vivo* in a better outcome in carriers of this genotype with advanced squamous head and neck carcinoma, and receiving chemo-radiotherapy [55]. However, these effects of the G allele may be reversed by a somatic p53 mutation on this allele [56]. Retention of the G allele with loss of the C allele in the tumor tissue has been associated with reduced survival in GC heterozygous BC patients [57].

It has been shown in various *in vitro* and mouse experiments that cell cycle arrest or apoptosis induced by radiotherapy and various chemotherapeutic drugs depends on an intact *TP53* pathway [58,59]. Preclinical studies suggested p53-dependent anthracycline-induced apoptosis and p53-independent taxane activity [58,60,61]. Other investigations have presented data in support of reduced anthracycline activity in *TP53* mutated tumors [62,63]. Thus, the presence of variations in the *TP53* gene could be one of the underlying causes of drug resistance, the major cause of treatment failure and cancer death.

We are aware of certain limitations of this study, such as: i) the different age distribution among cases and controls, ii) the non-homogenous proportion of patients with neoadjuvant and adjuvant treatment, iii) the wide range of therapeutic agents used. BC risk is also tightly linked to non-genetic factors such as pregnancy and hormonal history. Unfortunately, we were not able to assemble this information. Other variables such as age at menarche, oophorectomy, pregnancy and lactation history were also not included for comparison between cases and controls, since they were not available for both groups. These limitations may be counterbalanced by the homogeneity of the population and the detailed follow-up. To overcome the possible effect of age, we have repeated the analyses selecting patients and controls by matching for ±5 years. After matching, 570 patients (median age 56 years, range 27–71) and 575 controls (median age 51 years; range 33–91) were included in the analyses. For all the analyses performed, we obtained similar results for both study groups (whole unmatched and matched case-control group) (**S1 File**).

For the three studied polymorphisms, we also applied the Dunn-Bonferroni correction for multiple comparisons. After correction, the new adjusted threshold of P-value significance is 0.02 for analyses of individual SNPs. The rs1042522 polymorphism still appeared to be an independent prognostic marker in breast cancer patients.

In conclusion, in our study conducted on a Czech population of Caucasian origin, we reported for the first time that the most frequent *TP53* haplotype, previously associated with an increased CRC and pancreatic cancer risks, was also associated with an increased BC susceptibility. These results suggest that a particular genetic background based on certain haplotypes in relevant genes such as *TP53* may play a role in the susceptibility of solid cancers, as demonstrated on gastrointestinal cancers and BC. Importantly, we have also observed that both individual polymorphisms or specific haplotypes of the *TP53* gene were associated with BC clinical outcome.

Further studies are needed to replicate these findings in independent and larger populations to support the hypothesis that SNPs and haplotypes in *TP53* may be reliable predictive biomarkers in solid tumors and may help in directing to a better pre-selection of patients according to administered chemotherapeutic agents. Moreover, it is important to characterize functional relevance of the identified genetic variants and to elucidate their biologic associations.

## Supporting Information

S1 File Table A: Characteristics of therapy and clinical outcome of patients. Table B: Description of the breast cancer (BC) study group. Table C: Chemotherapy and hormonal therapy regimens of breast cancer (BC) patients with complete follow-up. Table D: Characteristics significantly affecting Overall Survival (OS) and Disease-Free Survival (DFS) in breast cancer (BC) patients (Multivariate Cox regression). Table E: Genotype distribution of the investigated *TP53* polymorphisms between matched breast cancer (BC) patients and controls. (DOCX)
