# Supplementary material for: Genotype and Haplotype Analyses of TP53 Gene in Breast Cancer Patients: Association with Risk and Clinical Outcomes
Source: PLoS One. 2015 Jul 30;10(7):e0134463. doi: 10.1371/journal.pone.0134463 (PMC4520609; doi:10.1371/journal.pone.0134463)
Supplement: S1 File — (DOCX) [file pone.0134463.s001.docx]

**Table A:** Characteristics of therapy and clinical outcome of patients

| **Parameter** | **Patients (%)** |
| --- | --- |
| **Neoadjuvant therapy**  Yes  No  N/A | 144 (20.4)  529 (75.0)  32 (4.6) |
|  |  |
| **Adjuvant Chemotherapy**  Yes  No  N/A | 370 (52.5)  291 (41.3)  44 (6.2) |
|  |  |
| **Radiotherapy**  Yes  No  N/A | 370  291  44 |
|  |  |
| **1^st^ line Hormonal therapy**  Yes  No  N/A | 218 (85.1)  37 (14.8)  1 (0.0001) |
|  |  |
| **Prognosis**  Alive  With progression  Without progression  Deceased  Deceased after progression | 528 (74.9)  78 (11.1)  450 (63.8)  77 (10.9)  39 (5.5) |
|  |  |
| **median OS** | 54.3 months |
|  |  |
| **median DFS** | 52.2 months |

N/A – not available; OS - overall survival; DFS – disease-free survival

**Table B:** Description of the breast cancer group

| **Characteristics** | **Patients**  **N (%)** |
| --- | --- |
|  |  |
| **Median Age±SD (years)** | 58.75±11.14 |
|  |  |
| **Menopause**  pre  peri  post  N/A | 129 (18.3)  22 (3.1)  499 (70.8)  55 (7.8) |
|  |  |
| **Tumor type**  Invasive ductal  Others (*in situ*, lobular, mucinous)  N/A | 530 (75.2)  160 (22.7)  15 (2.1) |
|  |  |
| **Breast cancer family history**  Positive  Negative  N/A | 180 (25.5)  497 (70.4)  33 (4.1) |
|  |  |
| **Ovarian cancer family history**  Positive  Negative  N/A | 10 (1.4)  667 (94.6)  27 (4) |
|  |  |
| **Estrogen receptor**  Positive  Negative  N/A | 499 (70.8)  195 (27.7)  11 (1.5) |
|  |  |
| **Progesterone receptor**  Positive  Negative  N/A | 465 (66)  227 (32.2)  18 (1.8) |
|  |  |
| **ERBB2**  Positive  Negative  N/A | 144 (20.4)  492 (69.8)  69 (9.8) |
|  |  |
| **pT**  1  2  3  4  **pTx** | 436 (61.8)  197 (27.9)  17 (2.4)  12 (1.7)  43 (6.2) |
|  |  |
| **pN**  0  1  2  3  **pNx** | 400 (56.7)  184 (26.1)  33 (4.1)  11 (1.6)  77 (11.5) |
|  |  |
| **cM**  0  1  N/A | 497 (70.5)  11 (1.6)  197 (27.9) |
|  |  |
| **Grade**  1  2  3  4  N/A | 109 (15.5)  273 (38.7)  201 (28.5)  54 (7.7)  68 (9.6) |
|  |  |

N/A – not available

**Table C:** Chemotherapy and hormonal therapy regimens of breast cancer patients with complete follow up

| **Characteristics** | **Type** | **n**^a^ | **%** |
| --- | --- | --- | --- |
| **Adjuvant or palliative regimen (n=370)** | Anthracycline alone  Taxane alone  Anthracycline & Taxane  Others | 192  93  34  44 | 51.9  25.1  9.2  11.9 |
| **Hormonal regimen only (n=218)** | Tamoxifen only  Aromatase inhibitors only  Others | 167  47  4 | 76.6  21.6  1.8 |

^a^Information about regimen was not available in 7 patients from the pre-treatment group (adjuvant regimens).

**Table D:** Characteristics significantly affecting Overall Survival (OS) and Disease-Free Survival (DFS) in breast cancer (BC) patients (Multivariate Cox regression)

|  | **OS** | | | **DFS** | | |
| --- | --- | --- | --- | --- | --- | --- |
|  | **HR** | **95%CI** | **P** | **HR** | **95%CI** | **P** |
| **Age**  >60 vs. < 60 years old | 1.03 | 1.01-1.06 | **0.006** | 1.03 | 1.01-1.05 | **0.002*** |
|  |  |  |  |  |  |  |
| **Hormonal therapy**  Yes vs. no | 1.93 | 1.15-3.25 | **0.01** | 1.70 | 1.10-2.62 | **0.02*** |
|  |  |  |  |  |  |  |
| **Radiotherapy**  Yes vs. no | 1.87 | 1.11-3.15 | **0.02** | 1.60 | 1.03-2.49 | **0.04** |
|  |  |  |  |  |  |  |
| ***TP53* rs1042522**  G/G vs. others | 0.78 | 0.49-1.25 | 0.30 | 0.65 | 0.44-0.96 | **0.03** |

HR, hazard ratio; 95% CI, confidence interval. Significant results in bold; significant differences after Dunn–Bonferroni correction (P<0.02) are marked with an asterisk.

**Table E.** Genotype distribution of the investigated *TP53* polymorphisms between matched breast cancer patients and controls

| **Genotype** | **Controls^a^** | **Cases^a^** | **OR^b^** | **95% CI** | **P** | **HWE^c^** |
| --- | --- | --- | --- | --- | --- | --- |
|  | **(n=575)** | **(n=570)** |  |  |  | Χ^2^, **P** |
| **rs17878362^d^** |  |  |  |  |  | 1.55, 0.46 |
| *A_1_A_1_* | 370 | 387 | REF |  |  |  |
| *A_1_A_2_* | 152 | 133 | 0.84 | 0.64-1.10 | 0.21 |  |
| *A_2_A_2_* | 10 | 12 | 1.15 | 0.49-2.69 | 0.75 |  |
| *A_1_A_2_+ A_2_ A_2_* | 162 | 146 | 0.89 | 0.68-1.16 | 0.38 |  |
| **rs1042522** |  |  |  |  |  | 2.44, 0.30 |
| *GG* | 259 | 302 | REF |  |  |  |
| *GC* | 235 | 223 | 0.81 | 0.64-1.04 | 0.11 |  |
| *CC* | 38 | 40 | 0.90 | 0.56-1.45 | 0.67 |  |
| *GC+CC* | 273 | 263 | 0.86 | 0.68-1.09 | 0.22 |  |
| **rs12947788** |  |  |  |  |  | 0.15, 0.93 |
| *CC* | 459 | 473 | REF |  |  |  |
| *CT* | 70 | 87 | 1.21 | 0.86-1.69 | 0.28 |  |
| *TT* | 2 | 2 | 0.97 | 0.14-6.92 | 0.98 |  |
| *CT+ TT* | 72 | 89 | 1.24 | 0.88-1.75 | 0.22 |  |

^a^Numbers may not add up to 100% of subjects due to genotyping failure. All samples that did not give a reliable result in the first round of genotyping were resubmitted to up to two additional rounds. Data points that were still not filled after this procedure had been left blank.

^b^Logistic regression analysis values are adjusted for age.

^c^X^2^ and P-values for the deviation of observed and the numbers expected from the Hardy-Weinberg equilibrium (HWE) in the controls.

^d^Allele A_2_ carries the 16-bp insertion within intron 3.

OR, odds ratio; CI, confidence interval.
